# Tissue Kallikrein as a Mediator in Stroke Outcomes among Patients With Metabolic Syndrome: A Multicenter Study Integrating Big Data and Biomarkers

**DOI:** 10.1002/mco2.70506

**Published:** 2025-11-26

**Authors:** Hang Ruan, Xiao Ran, Ting‐ting Xu, Da‐yong Li, Fang luo, Shu‐sheng Li, Dao‐Wen Wang, Qin Zhang

**Affiliations:** ^1^ Department of Critical‐Care Medicine Tongji Hospital Tongji Medical College Huazhong University of Science and Technology Wuhan China; ^2^ Department of Emergency Medicine Tongji Hospital Tongji Medical College Huazhong University of Science and Technology Wuhan China; ^3^ Department of Anesthesiology Hubei Key Laboratory of Geriatric Anesthesia and Perioperative Brain Health and Wuhan Clinical Research Center For Geriatric Anesthesia Tongji Hospital Tongji Medical College Huazhong University of Science and Technology Wuhan China; ^4^ The Institute of Hypertension and Department of Internal Medicine Tongji Hospital Tongji Medical College Huazhong University of Science and Technology Wuhan China

**Keywords:** big data, bioinformatic, KLK1, machine learning, metabolic syndrome, observational studies, stroke

## Abstract

Metabolic syndrome (MetS) poses a significant risk to the cerebrovascular system, impacting the prognosis of stroke patients. This study investigated the link between MetS and stroke‐related outcomes, exploring tissue kallikrein 1 (KLK1) as a potential mediator. In the derivation cohort, a total of 17,106 stroke‐diagnosed patients were assessed, with 6917 individuals (40.4%) presenting comorbid MetS. Multifactorial analysis identified stroke concurrent with MetS (adjusted odds ratio = 1.42; 95% confidence interval: 1.17–1.72; *p* < 0.001) as a risk factor for unfavorable outcomes among stroke patients. Further bioinformatics analyses indicated that obesity, diabetes, and hypertension were associated with reduced *KLK1* levels (all *p* < 0.05). In the validation cohort, 1268 first‐ever stroke patients were enrolled, confirming a higher incidence of adverse outcomes in those with MetS, compared with those without (223 (28.1%) vs. 167 (35.2%); *p* < 0.01). Stroke patients with MetS, exhibited lower KLK1 levels (16.8 ± 6.52 vs. 15.6 ± 6.3; *p* < 0.01). Mediation analyses supported that MetS contributed to adverse outcomes through the mediating effect of decreased KLK1 levels (*p* < 0.05). This study highlights the risk of MetS in stroke patients and suggests a potential role for KLK1 as a mediating factor.

## Graphical abstract

Metabolic syndrome is associated with adverse stroke outcomes. KLK1 acts as a mediating factor linking metabolic syndrome and stroke prognosis. KLK1 could be a therapeutic target for stroke patients with metabolic comorbidities.

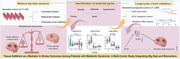

## 1 Introduction

Metabolic syndrome (MetS) is a cluster of metabolic abnormalities closely linked to insulin resistance, chronic inflammation, genetic factors, and characterized by obesity, abnormal glucose (Glu) metabolism, lipid disturbances, and hypertension [1]. The collective impact of these MetS features leads to systemic metabolic dysfunction, heightening the risk of severe complications such as cardiovascular and cerebrovascular diseases, posing a global health threat. With the global aging demographics and escalating overweight and obesity rates, the incidence of MetS is expanding worldwide. In the United States, statistics from 1999 to 2014 reflect an escalating trend in MetS prevalence (from 27.6 to 32.3%; adjusted odds ratio (aOR) (95% confidence interval (CI)) = 1.71 (1.42–2.05); *p *< 0.001) [2]. In China, data from the Chinese Centre for Disease Control and Prevention reveal a MetS prevalence of 33.9% among Chinese adults, affecting approximately 454 million individuals [3].

Theoretically, MetS and its components (obesity, hypertension, fasting blood Glu, triglycerides [TGs], and high‐density lipoprotein cholesterol) are intricately linked to the pathogenesis and pathophysiology of stroke. Individuals with chronic MetS status in the China Longitudinal Study of Health and Retirement cohort demonstrated elevated risks of cardiovascular disease (OR (95% CI) = 1.63 (1.25–2.13)) and stroke (OR (95% CI) = 2.95 (2.11–4.15)) in comparison with those without MetS [4]. However, diverging viewpoints exist, with some researchers suggesting a weak or insignificant relationship between MetS and stroke incidence and mortality. For instance, a prospective cohort study in the Mediterranean region over 13 years highlighted that the MetS components of hypertension (hazard ratio (HR) (95% CI) = 2.16 (1.15–4.06)) and increased fasting Glu (HR (95% CI) = 1.55 (1.00–2.40)) were independently linked to stroke risk, while indicating no substantial association between MetS and stroke risk (HR (95% CI) = 1.16 (0.76–1.76)) [5].

With the absence of a consensus in the existing literature regarding the prognostic implications of MetS in stroke, this investigation aimed to evaluate the influence of MetS on stroke patient prognosis, as well as explore the potential mediating molecules contributing to this impact. The study encompassed a retrospective cohort of 17,106 individuals alongside a multicenter prospective cohort of 1268 individuals. Initially, the analysis involved examining the influence of MetS on stroke patient prognosis through a comprehensive evaluation of data from the retrospective cohort. Subsequently, bioinformatics techniques were employed to identify potential molecules involved in the prognostic impact of various MetS components on stroke outcomes. The association between MetS, molecular expression, and prognosis was then validated in a prospective multicenter cohort study. The study postulated that MetS heightens the risk of adverse outcomes in stroke patients by downregulating tissue kallikrein 1 (KLK1) levels. By linking the clinical effects of MetS to poor stroke prognosis and potential molecular alterations, this study establishes a connection that bridges the clinical and molecular realms.

## 2 Results

### 2.1 Demographic and Clinical Characteristics of the Study Population

**FIGURE 1:**
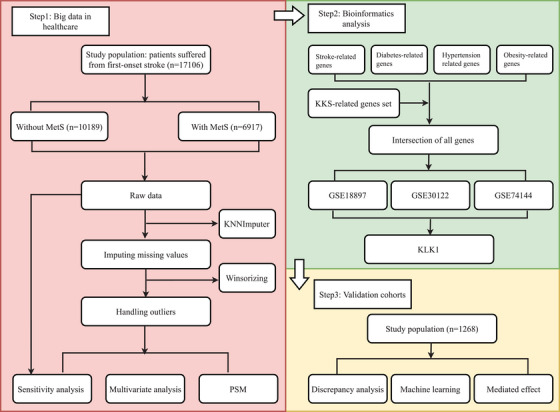
Flowchart of study design. The figure depicts a flowchart illustrating the study design, consisting of two cohorts: a retrospective cohort sourced from a genuine clinical environment and a national multicenter prospective cohort. The flowchart initially outlines the retrospective cohort study, starting with the initial pool of stroke patients, screening for inclusion/exclusion criteria, and the study objectives—to assess the impact of metabolic syndrome (MetS) on the prognosis of stroke patients and utilize bioinformatics analysis to identify potential causative molecules. It then delineates the subsequent step of revisiting a previously reported prospective cohort to reanalyze the associations between MetS, the potential causative molecules identified, and adverse outcomes following stroke admission, while also exploring potential mediating effects among these variables. KLK1, kallikrein 1; KKS, kallikrein–kinin system; MetS, metabolic syndrome; PSM, propensity score matching.

Figure 1 illustrates the flowchart of participant inclusion and exclusion processes for the present study. The retrospective cohort analyzed in the initial phase of this study comprised a total of 17,106 stroke patients, encompassing 8874 individuals with intracerebral hemorrhage (ICH) and 11,781 with ischemic stroke (IS) (including 3549 patients with both ICH and IS). Table 1 provides an overview of the demographic characteristics, behavioral habits, and metabolism‐related indicators of the study cohort. The study population was predominantly elderly (*n* = 13,101, 76.6%) with a minority being females (*n* = 6033, 35.3%). Among the patients, 5518 (32.3%) were smokers, and 3784 (22.1%) were drinkers. Adverse outcomes manifested in 745 patients (4.4%). Patients were stratified into groups based on the presence of comorbid MetS. Significant variations in age, body mass index (BMI), diastolic blood pressure (DBP), low‐density lipoprotein cholesterol (LDL‐C), total cholesterol (TC), Glu (Glu), and TGs were observed between the groups (all *p *< 0.05). Furthermore, significant differences existed between the two groups in terms of prevalent comorbidities such as hypertension, diabetes, chronic heart disease (CHD), and chronic respiratory disease (CRD) (all *p *< 0.001). Moreover, distinctions were noted in the utilization of therapeutic medications between the two groups (all *p *< 0.001).

**TABLE 1:** Baseline patient characteristics of individuals with stroke in the derivation cohort.

Characteristics	Without MetS (*N* = 10,189)	With MetS (*N* = 6917)	Overall (*N* = 17,106)	*p* Value
Patient status				
Sex female, %	3556 (34.9%)	2477 (35.8%)	6033 (35.3%)	0.222
Age ≥ 60, years	7641 (75.0%)	5460 (78.9%)	13,101 (76.6%)	**<0.001**
Smoker, %	3234 (31.7%)	2284 (33.0%)	5518 (32.3%)	0.079
Drinker, %	2187 (21.5%)	1597 (23.1%)	3784 (22.1%)	**0.012**
BMI ≥ 25, kg/m^2^	1877 (18.4%)	4936 (71.4%)	6813 (39.8%)	**<0.001**
Alb, g/L	40.5 ± 6.28	40.4 ± 6.19	40.4 ± 6.25	0.228
ICH, %	5206 (51.1%)	3668 (53.0%)	8874 (51.9%)	**0.013**
IS, %	6346 (62.3%)	4435 (64.12%)	11,781 (63.0%)	**<0.001**
MetS‐related indicators				
DBP, mmHg	82.0 ± 13.7	84.8 ± 14.7	83.1 ± 14.2	**<0.001**
SBP, mmHg	140 ± 23.2	139 ± 23.2	140 ± 23.2	0.672
LDL‐C, mg/dL	91.0 ± 29.8	99.8 ± 37.9	94.5 ± 33.6	**<0.001**
TC, mg/dL	150 ± 35.2	163 ± 46.6	155 ± 40.7	**<0.001**
HDL‐C, mg/dL	40.2 ± 12.0	39.9 ± 11.8	40.1 ± 12.0	0.193
TG, mg/dL	93.0 (69.1, 124.9)	148.0 (101.0, 213.5)	109.0 (77.1, 157.7)	**<0.001**
Glu, mg/dL	96.3 (88.0, 109.3)	131.9 (110.5, 174.4)	105.6 (91.4, 136.3)	**<0.001**
Comorbidities				
Diabetes, *n* (%)	708 (6.9%)	3812 (55.1%)	4520 (26.4%)	**<0.001**
Hypertension, *n* (%)	5229 (51.3%)	5610 (81.1%)	10,839 (63.4%)	**<0.001**
CHD, *n* (%)	3322 (32.6%)	2853 (41.2%)	6175 (36.1%)	**<0.001**
CRD, *n* (%)	4318 (42.4%)	3287 (47.5%)	7605 (44.5%)	**<0.001**
Treatment				
Aspirin use, *n* (%)	4426 (43.4%)	3441 (49.7%)	7867 (46.0%)	**<0.001**
Clopidogrel use, *n* (%)	3177 (31.2%)	2588 (37.4%)	5765 (33.7%)	**<0.001**
Statin use, *n* (%)	5498 (54.0%)	4338 (62.7%)	9836 (57.5%)	**<0.001**
Clinical outcomes				
Adverse outcome, *n* (%)	337 (3.3%)	408 (5.9%)	745 (4.4%)	**<0.001**
Need for surgery, *n* (%)	380 (3.7%)	262 (3.8%)	642 (3.8%)	0.844
Need for IMV, *n* (%)	377 (3.7%)	409 (5.9%)	786 (4.6%)	**<0.001**
Need for vasoactive drugs, *n* (%)	1188 (11.7%)	1253 (18.1%)	2441 (14.3%)	**<0.001**

*Note*: Adverse outcomes, designated as the primary endpoint, were defined as a composite event encompassing stroke‐related mortality and stroke recurrence.​.

Data are presented as median (IQR) for non‐normal continuous variables, mean ± SD for normal continuous variables, and *n* (%) for categorical variables. The table presents intergroup comparisons between the stroke without MetS and stroke with MetS groups. For continuous variables, if the data were normally distributed but did not satisfy the homogeneity of variance assumption, the Welch's *t*‐test was applied. If the data were not normally distributed, the Wilcoxon rank‐sum test was used. For categorical variables, the chi‐square test was employed. Bold *p* values (*p *< 0.05) indicate significant between‐group differences.

Abbreviations: Alb, albumin; BMI, body mass index; CHD, chronic cardiac disease; CRD, chronic respiratory disease; DBP, diastolic blood pressure; Glu, glucose; HDL‐C, high‐density lipoprotein cholesterol; ICH, intracerebral hemorrhage; IMV, invasive mechanical ventilation; IS, ischemic stroke; LDL‐C, low‐density lipoprotein cholesterol; MetS, metabolic syndrome; SBP, systolic blood pressure; TC, total cholesterol; TG, triglyceride.

### 2.2 MetS was an Independent Risk Factor for Adverse Outcome

Univariate and multivariate logistic regression models were employed to identify risk factors contributing to adverse outcomes in stroke patients (Table 2). In the initial univariate analysis, significant associations with poor prognosis in stroke patients were observed for factors including MetS, male gender, BMI (≥25 kg/m^2^), DBP, LDL‐C, TC, Glu, CHD, CRD, as well as nonuse of aspirin, clopidogrel, and statins (*p* < 0.05). Subsequent multifactorial analyses were conducted by adjusting for these potential confounders. The results confirmed that the combination of stroke and MetS (OR (95% CI) = 1.42 (1.17–1.72), *p* < 0.001) emerged as an independent risk factor for adverse outcomes.

**TABLE 2:** Univariate and multivariate analyses for the adverse outcome in the derivation cohort.

Characteristics	Univariate analysis			Multivariate analysis	
	Odds ratio (95% CI)	*p* Value		Odds ratio (95% CI)	*p* Value
MetS (no vs. yes)	1.83 (1.58–2.12)	<0.001		1.42 (1.17–1.72)	<0.001
Age (<60 vs. ≥60, years)	0.98 (0.81–1.14)	0.622			
Sex (female vs. male)	1.53 (1.30–1.81)	<0.001		1.59 (1.33–1.89)	<0.001
Smoker (no vs. yes)	1.12(0.96–1.30)	0.157			
Drinker (no vs. yes)	1.15(0.96–1.36)	0.121			
BMI (<25 vs. ≥25, kg/m^2^)	1.59 (1.37–1.84)	<0.001		1.19 (0.97–1.45)	0.960
Alb, g/L	1.00 (0.99–1.01)	0.806			
DBP, mmHg	0.97 (0.97–0.98)	<0.001		0.98 (0.98–0.99)	<0.001
SBP, mmHg	1.00 (1.00–1.00)	0.867			
LDL‐C, mg/dL	0.99 (0.99–0.99)	<0.001		0.99 (0.99–1.00)	<0.001
TC, mg/dL	0.99 (0.99–1.00)	<0.001		1.00 (1.00–1.00)	0.463
HDL‐C, mg/dL	1.00 (1.00–1.01)	0.125			
TG, mg/dL	1.00 (1.00–1.00)	0.159			
Glu, mg/dL	1.01 (1.01–1.01)	<0.001		1.00 (1.00–1.01)	<0.001
Diabetes (no vs. yes)	1.03 (0.87–1.22)	0.725			
Hypertension (no vs. yes)	0.95 (0.82–1.11)	0.531			
CHD (no vs. yes)	2.88 (2.48–3.35)	<0.001		2.69 (2.28–3.17)	<0.001
CRD (no vs. yes)	4.03 (3.40–4.78)	<0.001		2.94 (2.47–3.51)	<0.001
Aspirin use (no vs. yes)	0.50 (0.42–0.58)	<0.001		0.78 (0.62–0.97)	<0.001
Clopidogrel use (no vs. yes)	0.58 (0.48–0.68)	<0.001		0.86 (0.69–1.09)	0.216
Statin use (no vs. yes)	0.46 (0.40–0.54)	<0.001		0.44 (0.35–0.55)	<0.001

*Note*: Adverse outcomes, designated as the primary endpoint, were defined as a composite event encompassing stroke‐related mortality and stroke recurrence.

The table identifies independent factors associated with adverse outcome via logistic regression. The outcome variable is coded as [1 = event occurrence, 0 = no event], and independent variables include patient status, mets‐related indicators, comorbidities, and treatment. First, univariate binary logistic regression was performed via the generalized linear model, with odds ratios (OR) and 95% confidence intervals (95%CI) calculated for each variable. Variables with *p* value < 0.1 in univariate analysis were included in multivariate binary logistic regression, with model‐related tests conducted.

Abbreviations: Alb, albumin; BMI, body mass index; CHD, chronic cardiac disease; CRD, chronic respiratory disease; DBP, diastolic blood pressure; Glu, glucose; HDL‐C, high‐density lipoprotein cholesterol; ICH, intracerebral hemorrhage; IMV, invasive mechanical ventilation; IS, ischemic stroke; LDL‐C, low‐density lipoprotein cholesterol; MetS, metabolic syndrome; SBP, systolic blood pressure; TC, total cholesterol; TG, triglyceride.

**FIGURE 2:**
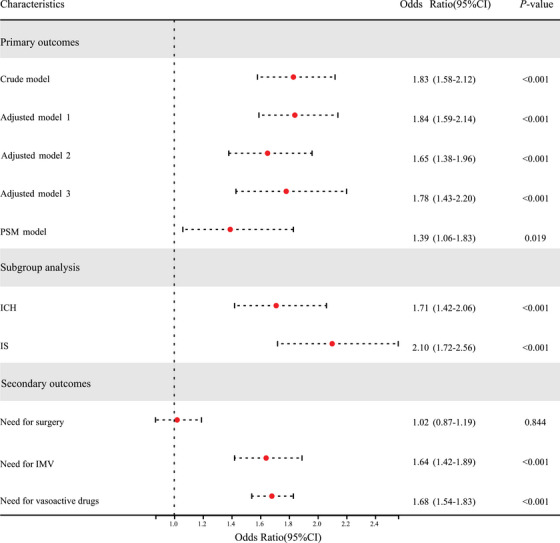
Effect of metabolic syndrome (MetS) on clinical outcomes. The first section presents analyses of the primary outcome (adverse outcome), with robustness verified across four models: crude model (unadjusted for any covariates); adjusted model 1 (adjusted for age and sex); adjusted model 2 (adjusted for sex, age, body mass index, smoking, and alcohol consumption); and adjusted model 3 (fully adjusted for age, alcohol consumption, body mass index, diastolic blood pressure, low‐density lipoprotein cholesterol, total cholesterol, triglycerides, glucose, diabetes, hypertension, chronic cardiac disease, chronic respiratory disease, aspirin use, clopidogrel use, and statin use). Additionally, propensity score matching (PSM) was applied to harmonize baseline data between the two comparison groups, with variables for PSM screened via directed acyclic graphs. The second section shows subgroup analyses, stratifying patients by stroke type (ischemic stroke and intracerebral hemorrhage) to explore the consistency of MetS's association with the primary outcome across subgroups. The third section presents the analysis of MetS's influence on secondary outcomes, with effect sizes in the plot expressed as odds ratios (OR) and 95% confidence intervals (95%CI); the vertical line represents OR = 1 (no association), and *p* values denote statistical significance. CI, confidence intervals; ICH, intracerebral hemorrhage; IMV, invasive mechanical ventilation; IS, ischemic stroke; OR, odds ratios; PSM, propensity score matching. *Adverse outcomes*, designated as the primary endpoint, were defined as a composite event encompassing stroke‐related mortality and stroke recurrence. *Need for surgery*: Defined as the medically necessary performance of surgical procedures aimed at managing stroke‐related complications or progressive pathological conditions, excluding prophylactic or nonstroke‐related surgical interventions.​ *Need for vasoactive drugs*: Refers to the clinical decision to administer pharmaceutical agents that modulate vascular tone or cardiac function (e.g., vasopressors such as norepinephrine, inotropes such as dobutamine) for the purpose of maintaining hemodynamic stability in stroke patients.​ *Need for invasive mechanical ventilation*: Characterized by the requirement for positive‐pressure ventilation delivered via an artificial airway (e.g., endotracheal intubation, tracheostomy) to address stroke‐induced respiratory dysfunction.

The effect size of MetS and primary outcomes was further investigated in the study with appropriate adjustments for potential confounders (Figure 2). Table S1 presents the multicollinearity analysis results for the variables in the various adjusted models (all VIF < 10). In the unadjusted model, the logit regression model corrected for confounders, and the propensity score matching (PSM) model (Table S2 and Figure S1), the significant association between MetS and adverse outcomes persisted (all model OR > 1; *p* < 0.05). Within the subgroups of ICH and cerebral IS, MetS emerged as a risk factor for adverse outcomes in stroke patients (ICH: OR (95% CI) = 1.71 (1.42–2.06), *p* < 0.001; IS: OR (95% CI) = 2.10 (1.72–2.56), *p* < 0.001). Moreover, in the examination of secondary outcomes, patients with concurrent MetS exhibited an increased likelihood of requiring mechanical ventilation and vasoactive drug support therapy (all OR > 1; *p* < 0.001). Notably, no significant association was observed between comorbid MetS and the necessity for surgical intervention (OR (95% CI) = 1.02 (0.87–1.19), *p* = 0.844). In the sensitivity analysis, the study obtained consistent results when reanalyzing the raw data (Table S3). The approximate *E*‐value for unmeasured confounding was 2.04 for the point estimate and 1.83 for CI (Figure S2).

### 2.3 *KLK1* as a Potential Molecular Associated with Stroke and MetS Components

**FIGURE 3:**
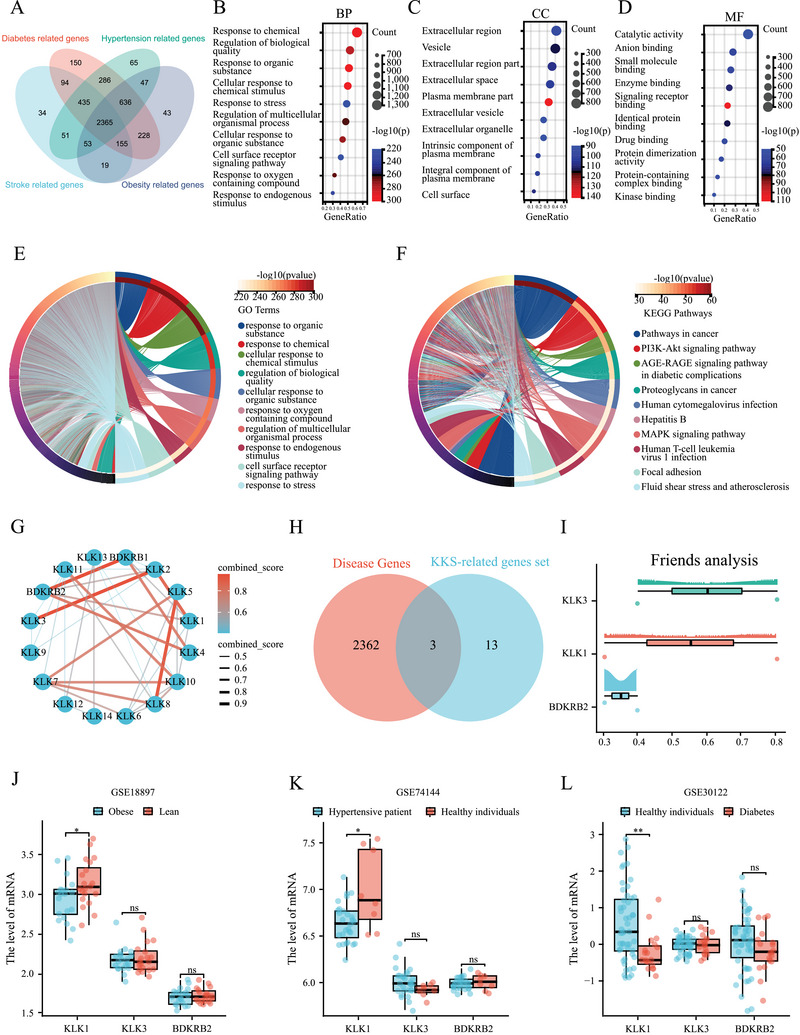
The workflow for identifying kallikrein–kinin system (KKS)‐related genes and analyzing their associations with metabolic and cerebrovascular diseases. (a) A Venn diagram was used to visualize the intersection of genes associated with obesity, stroke, diabetes, and hypertension. (b) Enrichment analysis of Gene Ontology (GO) terms in the biological process (BP) category. (c) Enrichment analysis of GO terms in the cellular component (CC) category. (d) Enrichment analysis of GO terms in the Molecular function (MF) category. (e) Further GO enrichment analysis of the four‐disease intersecting genes to refine their functional annotations. (f) Kyoto Encyclopedia of Genes and Genomes (KEGG) enrichment analysis of the intersecting genes, exploring significantly enriched signaling pathways to provide insights into the molecular mechanisms linking these genes to diseases. (g) A protein–protein interaction (PPI) network constructed using the KKS‐related gene set, intuitively displaying the interaction relationships between genes and identifying core regulatory genes within the KKS. (h) Venn diagram showing the intersection between disease‐associated genes (obesity, stroke, diabetes, hypertension) and the KKS‐related gene set, verifying the potential association of KKS with these diseases. (j–l) Box plots visualizing the expression distribution of the KKS‐related gene KLK1 in three Gene Expression Omnibus (GEO) datasets, with each box representing the interquartile range (IQR) from the first quartile (Q1, 25th percentile) to the third quartile (Q3, 75th percentile), and the horizontal line inside each box indicating the median (50th percentile): GSE18897 (obese vs. lean: 2.94 ± 0.27 vs. 3.14 ± 0.29, *p* = 0.033), GSE74144 (hypertensive patients vs. healthy individuals: 6.64 ± 0.21 vs. 7.00 ± 0.42, *p* = 0.033), and GSE30122 (healthy vs. diabetic individuals: 0.34 (−0.17, 1.24) vs. −0.43 (−0.54, −0.04), *p* = 0.003), comparing KLK1 expression differences between disease and normal/healthy states. BP, biological process; CC, cellular component; GEO, Gene Expression Omnibus; GO, Gene Ontology; IQR, interquartile range; KEGG, Kyoto Encyclopedia of Genes and Genomes; KKS, kallikrein–kinin system; MF, molecular function; PPI, protein–protein interaction; Q1, first quartile; Q3, third quartile.

The gene sets associated with stroke, diabetes, hypertension, and obesity collectively comprised 2365 coexpressed genes (Figure 3A). Enrichment analysis revealed significant enrichment of molecular pathways such as stress and atherosclerosis, as well as cell surface receptor signalling pathways, in the intersecting genes (Figure 3B–F). A protein–protein interaction (PPI) network was constructed using the relevant genes within the kallikrein–kinin system (KKS)‐related genes to explore the core genes in this system (Figure 3G). Wayne plots depicted the presence of three common genes (*KLK1, KLK3, BDKRB2*; Figure 3H) among KKS‐related genes and disease‐related genes. Friends’ analysis indicated that *KLK1* exhibited moderate importance among these three genes (Figure 3I).

Detailed information about the GSE18897, GSE74144, and GSE30122 datasets was provided in Tables S4–S6 and Figure S3. Among these three gene sets, only *KLK1* displayed significant differences between the groups (GSE18897, obese vs. lean = 2.94 ± 0.27 vs. 3.14 ± 0.29, *p *< 0.05; GSE74144, hypertensive patients vs. healthy individuals = 6.64 ± 0.21 vs. 7.00 ± 0.42, *p* < 0.05; GSE30122, healthy vs. diabetic individuals = 0.55 ± 1.02 vs. −0.22 ± 0.52, *p* < 0.01; Figure 3J–L). Levels of *KLK3* and *BDKRB2* did not demonstrate significant differences in the analyzed gene sets. In this study, a disease enrichment analysis was conducted for differentially expressed genes. The results indicate that among the genes associated with stroke, only *KLK1* was deemed appropriate (Table S7).

### 2.4 Mediating Effect of MetS on Stroke via KLK1 in Validation Cohort

Table 3 presents the demographic characteristics of the validation cohort, consisting of 793 stroke patients without comorbid MetS and 475 stroke patients with comorbid MetS. A significant reduction in KLK1 expression levels was observed in patients with MetS compared with those without (15.6 ± 6.3 vs. 16.8 ± 6.52, *p* = 0.002). The incidence of adverse outcomes differed significantly between the two groups (35.2 vs. 28.1%, *p* = 0.009).

**TABLE 3:** Baseline characteristics of individuals in the prospective cohort study.

Characteristics	Without MetS (*N* = 793)	With MetS (*N* = 475)	Overall (*N* = 1268)	*p* Value
KLK1, µg/dL	16.8 ± 6.52	15.6 ± 6.3	16.3 ± 6.5	**0.002**
Type of disease				0.550
IS, %	593 (74.8%)	348 (25.2%)	941 (74.2%)	
ICH, %	200 (73.3%)	127 (26.7%)	327 (25.8%)	
Patient status				
Age ≥ 60, years	488 (61.5%)	291 (61.3%)	779 (61.4%)	0.992
Sex female, %	291 (36.7%)	199 (41.9%)	490 (38.6%)	0.066
BMI ≥ 25, kg/m^2^	238 (30.0%)	308 (64.8%)	546 (43.1%)	**<0.001**
Smoker, %	259 (32.7%)	132 (27.8%)	391 (30.8%)	0.069
MetS‐related indicators				
SBP, mmHg	146.1 ± 25.2	152.3 ± 24.1	148.4 ± 25.0	**<0.001**
DBP, mmHg	86.9 ± 14.6	90.2 ± 13.9	88.1 ± 14.4	**<0.001**
TC, mg/dL	186.1 ± 42.9	192.6 ± 48.8	188.5 ± 45.3	**0.017**
TG, mg/dL	115.2 (81.5, 147.1)	186.1 (155.2, 243.7)	141.8 (97.5, 194.9)	**<0.001**
HDL‐C, mg/dL	48.4 ± 16.6	42.4 ± 14.2	46.1 ± 16.0	**<0.001**
Glu, mg/dL	92.2 (81.9, 105.1)	120.8 (106.4, 140.3)	101.0 (85.8, 120.8)	**<0.001**
Comorbidities				
Hypertension, *n* (%)	407 (51.3%)	368 (77.5%)	775 (61.1%)	**<0.001**
Diabetes, *n* (%)	52.0 (6.6%)	89.0 (18.7%)	141 (11.1%)	**<0.001**
CHD, *n* (%)	158 (19.9%)	96.0 (20.2%)	254 (20.0%)	0.902
Adverse outcome, *n* (%)	223 (28.1%)	167 (35.2%)	390 (30.8%)	**0.009**

*Note*: Adverse outcomes, designated as the primary endpoint, were defined as a composite event encompassing stroke‐related mortality and stroke recurrence.

Data are presented as median (IQR) for non‐normal continuous variables, mean ± SD for normal continuous variables, and *n* (%) for categorical variables. The table presents intergroup comparisons between the stroke without MetS and stroke with MetS groups. For continuous variables, if the data were normally distributed but did not satisfy the homogeneity of variance assumption, the Welch's *t*‐test was applied. If the data were not normally distributed, the Wilcoxon rank‐sum test was used. For categorical variables, the chi‐square test was employed. Bold *p* values (*p *< 0.05) indicate significant between‐group differences.

Abbreviations: BMI, body mass index; CHD, chronic cardiac disease; DBP, diastolic blood pressure; Glu, glucose; HDL‐C, high‐density lipoprotein cholesterol; ICH, intracerebral hemorrhage; IS, ischemic stroke; KLK1, kallikrein 1; SBP, systolic blood pressure; TC, total cholesterol; TG, triglyceride.

**FIGURE 4:**
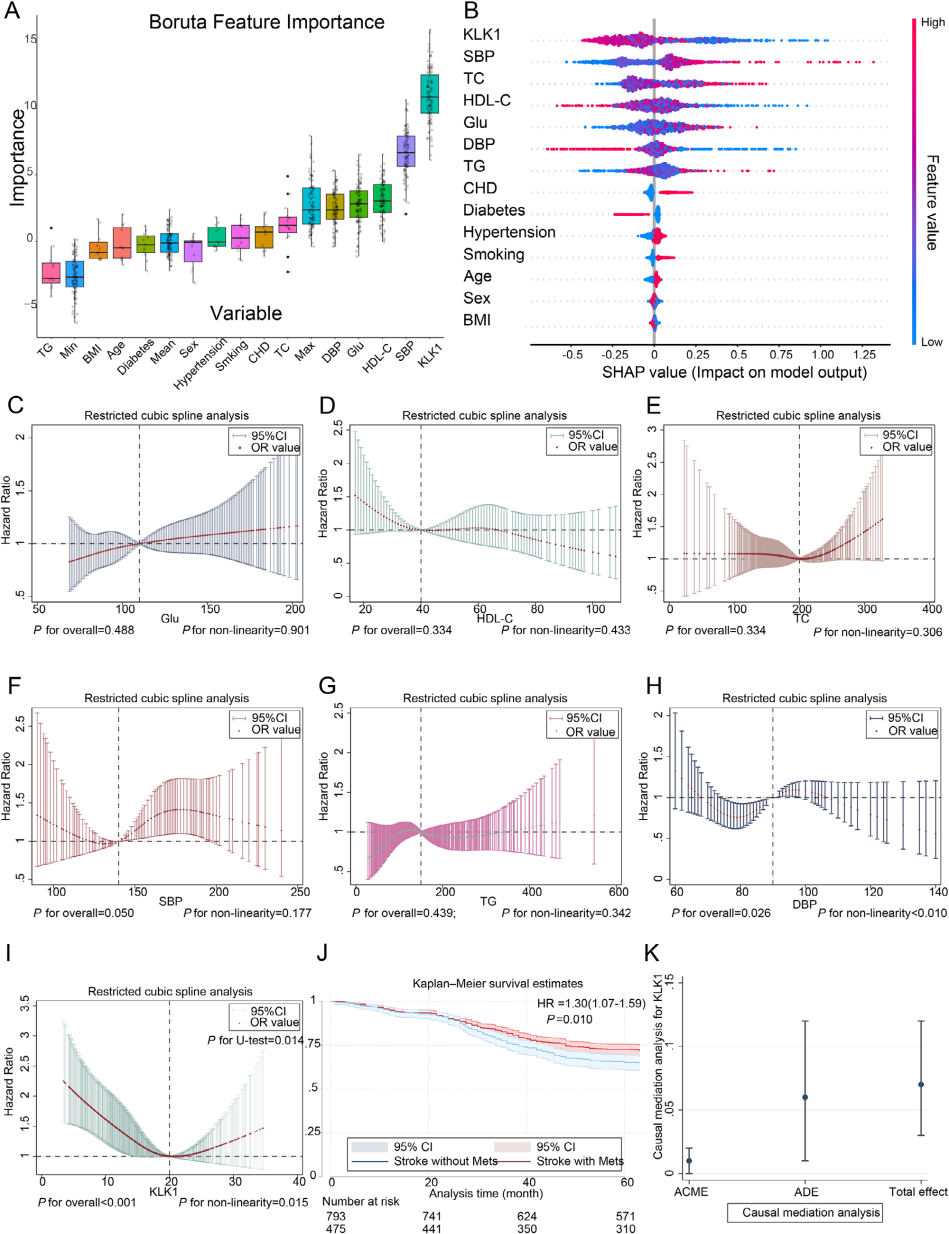
The correlation between MetS, KLK1, and clinical outcomes. Panel (a) depicts the feature importance ranking from the Boruta feature selection process, which was applied to identify the most clinically relevant variables (including MetS and KLK1) that contribute to predicting adverse clinical outcomes, with higher importance values indicating stronger relevance to the outcome. Panel (b) presents the SHapley Additive exPlanations (SHAP) summary plot for predictive features in the Cox proportional hazards model; this plot quantifies the contribution of each feature to the model's survival outcome predictions. Each row corresponds to one predictive feature, and each point represents a single patient sample. The horizontal position of points indicates the SHAP value (positive values increase predicted risk of adverse outcomes, while negative values decrease it), and the color gradient denotes feature value magnitude (red = high feature values, blue = low feature values). Panels (c–i) illustrate analyses of nonlinear associations between continuous variables and adverse outcomes using restricted cubic splines (RCS); these plots test whether the relationship between each continuous variable and the risk of adverse outcomes follows a nonlinear pattern rather than a simple linear one. Panel (j) shows Kaplan–Meier survival curves comparing stroke patients with MetS versus those without MetS; the curves visualize differences in survival probability over the follow‐up period, with the log‐rank test used to statistically assess whether MetS is associated with altered survival outcomes in stroke patients. Panel (k) displays results of causal mediation analysis for KLK1, which was conducted to evaluate whether KLK1 acts as a mediator in the relationship between MetS and adverse clinical outcomes—specifically testing if MetS influences adverse outcomes partially or fully through regulating KLK1 expression. Cox, Cox proportional hazards model; KLK1, kallikrein 1; MetS, metabolic syndrome; RCS, restricted cubic splines; SHAP, SHapley Additive exPlanations.

Utilizing the Boruta algorithm to evaluate the influence of various factors on the prognosis of stroke patients, the results revealed that KLK1 was ranked the highest (Figure 4A). Moreover, upon interpreting the outcomes with SHapley Additive exPlanations (SHAP), KLK1 was identified as the most significant contributor to the outcomes in the Cox proportional hazards regression model adjusted for confounders (Figure 4B). The RCS regression analysis indicated that Glu, SBP, HDL‐C, TC, and TG exhibited linear associations with outcome events (overall *p* > 0.05, nonlinearity *p* > 0.05; Figure 4C–G). Conversely, DBP and KLK1 displayed nonlinear associations with outcome events (overall *p* < 0.05, nonlinearity *p* < 0.05; Figure 4H,I). Furthermore, the *U*‐test suggested a U‐shaped association between KLK1 levels and outcome events, indicating that both excessively high and low levels of KLK1 influenced the prognosis of stroke patients (*p* for *U*‐test = 0.014; Figure 4I). In analyses stratifying patients by stroke type (ICH and IS), KLK1 demonstrated a nonlinear association with clinical outcomes for both ICH and IS (Figure S4).

The Kaplan–Meier plot demonstrated that stroke patients with comorbid MetS exhibited a poorer prognosis compared with those without (HR (95% CI) = 1.30 (1.07–1.59), log‐rank *p* values = 0.010, *p* for phtest > 0.05; Figure 4J). The mediation analysis revealed a statistically significant result (*p* < 0.05), with an estimated average causal mediation effect of 0.8 and a 95% CI of (0.2–1.5%) (Figure 4K).

## 3 Discussion

This study employed a comprehensive approach combining medical big data analysis, bioinformatics analysis, and cohort studies to investigate the association between MetS and adverse outcomes in cerebrovascular disease, as well as to explore potential mediating factors. Our findings revealed that stroke patients with comorbid MetS experienced worse outcomes compared with those without. Additionally, individuals with MetS exhibited significantly reduced levels of KLK1, and mediation analyses identified KLK1 as a potential mediator in the impact of MetS on stroke patients’ prognosis. These results suggest the potential for timely and proactive interventions targeting modifiable risk factors, such as measuring plasma KLK1 concentrations in individuals with MetS and stroke, to mitigate stroke risk and recurrence, thereby enhancing outcomes in cerebrovascular disease management.

Our conclusions find support in several past clinical studies. A meta‐analysis incorporating 13 cohort studies examined the association between MetS and its components with the risk of stroke recurrence and death. The study results indicated that MetS posed as a risk factor for stroke recurrence (relative risk (RR) 95% CI = 1.46 (1.07–1.97), *p* < 0.05) [6]. Another meta‐analysis encompassed five studies with 7752 stroke patients who had a history of stroke or transient ischemic attack, revealing that individuals with MetS had a notably higher risk of stroke recurrence compared with those without (RR (95% CI) = 1.52 (1.17–1.97), *p* < 0.05) [7]. Additionally, a 5‐year follow‐up study conducted in a Chinese population demonstrated that MetS heightened the risk of stroke, with an increased HR for IS (HR 95% CI = 5.1 (1.9–7.4), *p* < 0.05) and ICH (HR 95% CI = 3.3 (1.7–5.7), *p* < 0.05) [8].

The pathophysiological mechanisms through which MetS impacts stroke prognosis may involve endothelial cell dysfunction as a crucial link between these conditions. Patients with MetS frequently exhibit chronic inflammatory responses, hypertension, hyperglycemia, and lipid metabolism disorders, all of which can detrimentally affect endothelial cell function and contribute to endothelial dysfunction. For instance, hypertension can directly impair cerebrovascular endothelial cell structure and function, leading to elevated synthesis of vasoconstrictive substances like angiotensin II and suppression of vasodilatory factors. Moreover, endothelial injury caused by exfoliation, shear stress variations, or inflammation can trigger the onset of a stroke [9]. Elevated levels of advanced glycation end products (AGEs) seen in diabetic states can induce oxidative and inflammatory reactions in endothelial cells by interacting with the receptor for AGEs (RAGE). The activation of p38 and ERK1/2 in endothelial cells leads to decreased endothelial nitric oxide synthase (eNOS) expression, increased oxidative stress, and resultant endothelial dysfunction [10]. This dysfunction promotes the infiltration of LDL into the subendothelial layer, where it accumulates, oxidizes to ox‐LDL, and attracts inflammatory immune cells, contributing to chronic inflammation [10]. Endothelial dysfunction is marked by reduced vasodilation, cellular proliferation, platelet adhesion and activation, and a proinflammatory and prothrombotic state [11]. These changes further contribute to oxidative stress, inflammation, elevated vascular tone, blood–brain barrier impairment, and affect stroke prognosis. KLK1, abundantly present in human endothelial cells, exhibits potent angiogenic properties, mediates relaxation of human umbilical veins, and is a key factor in vascular homeostasis [12, 13]. Building upon these insights, our study introduces a novel hypothesis proposing that MetS impacts stroke prognosis through the downregulation of KLK1 levels.

The bioinformatics analysis in the current study indicates KLK1 as a potential molecule that connects various aspects of the MetS and unfavorable stroke outcomes. Within the hypertensive, diabetic, and obese gene sets, the levels of KLK1 were notably reduced in obese, diabetic, and hypertensive populations in comparison with individuals with normal health status. These findings from the bioinformatics analysis were corroborated by parallel basic research studies. First, the association between KLK1 and hypertension appears more evident in current investigations, as demonstrated by decreased KLK1 levels in hypertensive individuals. Notably, the submandibular gland and kidney of hypertensive rats exhibited significantly lower levels of glandular kallikrein compared with normotensive rats [14].

Moreover, the delivery of the human tissue kinin‐releasing enzyme gene effectively reduced systolic blood pressure in spontaneously hypertensive rats [15]. Second, it is suggested that KLK1 may interact with cholesterol, based on AutoDock analysis results indicating potential binding sites between cholesterol and the C‐terminal KLK1 and KLK2 sequences, the N‐terminal KLK7 and KLK12 sequences, as well as regions near the intermediate KLK8 sequence [16]. Additionally, KLK1 levels may be related to body weight regulation, as observed in KLK1‐deficient mice gaining less weight compared with wild‐type littermates when subjected to a high‐fat diet [17]. Nevertheless, the conclusions from current studies regarding whether KLK1 levels are increased or decreased in diabetic individuals are conflicting. One study reported lower KLK1 protein levels in patients with type 2 diabetes in the pancreatic islet cells, while the mRNA levels of *KLK1* were similar to those of healthy subjects [18]. In contrast, animal experiments indicated reduced expression of the rat KLK7 and the rat KLK1 in streptozotocin‐induced diabetic rats [19]. However, a separate study documented elevated levels of KLK1 in the pancreatic islet cells of diabetic individuals, with the expression of *KLK1* being upregulated in human diabetic renal tissues and induced by high Glu in human proximal tubular epithelial cells [20]. The discrepancies in these observations may be attributed to variations in KLK1 expression levels at different stages of diabetes mellitus. In summary, considering the MetS as a complex condition encompassing obesity, hypertension, and diabetes mellitus, it is plausible that a significant decrease in KLK1 may manifest in patients with the MetS.

Subsequently, this study delved into the relationship among MetS, KLK1 levels, and stroke prognosis in patients, drawing from an extensive multicenter prospective study conducted previously. Our study reveals a U‐shaped relationship between KLK1 levels and the prognosis of both hemorrhagic and ISs, indicating that both elevated and decreased KLK1 levels can negatively impact stroke patient outcomes. A deficiency of endogenous KLK1 has been linked with various vascular disorders, including hypertension, stroke, and myocardial infarction [21]. Supplementing KLK1 has demonstrated benefits in mitigating cerebral edema and inflammation, preventing apoptosis, and promoting neurogenesis and angiogenesis [21, 22]. Recently, we demonstrated that KLK1 supplementation during cerebral ischemia can inhibit the excessive activation of KKS system induced by reperfusion injury, thereby reducing blood–brain barrier damage and protecting the neurovascular unit [13]. Moreover, the administration of exogenous KLK1 enhances collateral circulation in the ischemic brain by generating novel bradykinin peptides that promote localized vasodilation of collateral vessels [23]. This process improves blood flow and reduces the risk of recurrent ISs. In the context of ICH, KLK1 has been shown to alleviate cerebral vasospasm in a rabbit model of subarachnoid hemorrhage and to inhibit endothelial cell apoptosis in the basilar artery [24]. In models of cerebral ischemia–reperfusion, KLK1 exhibits neuroprotective properties by upregulating the Nrf2 pathway and downregulating the TLR4/NF‐κB pathway [25].

However, despite these neuroprotective effects, elevated concentrations of KLK1 may initiate inflammatory cascades through interactions with protease‐activated receptors [26, 27, 28, 29]. In cases of ICH, hyperglycemia exacerbates hematoma expansion by stimulating plasma kinin‐releasing enzymes. Conversely, Streptozotocin‐induced diabetic Klkb1−/− mice, upon receiving autologous blood injections, show a significant reduction in hematoma volume. Furthermore, Klkb1−/− mice demonstrate a prolonged activated partial thromboplastin time [30]. We also found that high levels of KLK1 can enhance blood–brain barrier permeability. In vitro study results indicated that high doses of KLK1 decreased the integrity of the normal blood–brain barrier, reduced the expression of Zona Occludens 1, and upregulated the mRNA levels of Bradykinin 2 receptor (B2R) and eNOS, while also increasing the abundance of B2R [31]. These findings align with our earlier clinical observations suggesting that elevated KLK1 levels, within certain ranges, predict better stroke outcomes; however, excessively high KLK1 levels do not correlate with improved outcomes [26, 32]. Therefore, we propose that overly elevated KLK1 may exacerbate damage by increasing blood–brain barrier permeability through receptor‐mediated mechanisms. This observation indicates that varying concentrations of KLK1 can produce bidirectional effects, suggesting that KLK1 may elicit distinct and potentially opposing biological responses under different physiological conditions [33].

## 4 Limitations

While this study has provided insights into the impact of MetS on the prognosis of stroke patients from various angles, we acknowledge the inherent limitations of our research. First, due to the lack of uniform diagnostic criteria for MetS, there may exist objective variations in physical parameters such as height, weight, lipid levels, and waist circumference among different ethnic groups. To address this, our study adopted the diagnostic criteria specific to our country, considering the characteristics of our national population. In China, the Chinese Diabetes Society guidelines from 2004 suggest diagnosing overweight and/or obesity based on a BMI of 25 kg/m^2^or higher as a proxy for central obesity. This criterion was selected due to the prevalent distribution of body fat in the Chinese population, where a significant percentage of individuals exhibit abdominal obesity even at BMI values ranging from 24.0 to 27.9 kg/m^2^, and a substantial majority demonstrate abdominal obesity at a BMI of 28 kg/m^2^ or greater [34]. The absence of waist circumference data in our cohort constrained our capacity to adhere to the most current diagnostic criteria for MetS, representing another limitation of this study. Additionally, while we observed a connection between MetS and KLK1 levels, our study did not delve into the underlying biological mechanisms that could elucidate these associations. Future research endeavors should be bifurcated into two facets: first, further investigating the correlation between MetS, stroke, and KLK1 levels under varying diagnostic criteria for MetS; and second, conducting basic medical research to unravel the molecular mechanisms at play and explore the therapeutic potential of modulating KLK1 activity in the management of cerebrovascular disorders linked to MetS. Last, the utilization of transcriptomics data from the Gene Expression Omnibus (GEO) dataset inherently introduces challenges related to diverse ethnicities within the dataset, variations in testing standards, and inadequate sample sizes. This limitation is acknowledged in our study, and we intend to address it by collecting data from patients in future studies.

## 5 Conclusions

This exploratory study indicates that MetS influences the prognosis of stroke patients through the modulation of KLK1 levels. KLK1 may represent a promising therapeutic target; however, additional experimental validation is necessary to substantiate these findings.

## 6 Methods and Materials

### 6.1 Study Designs

This study comprises two cohorts: a retrospective cohort sourced from an authentic clinical environment and a national multicenter prospective cohort. Initially, a retrospective cohort study was conducted to investigate the influence of MetS on the prognosis of stroke patients. Bioinformatics analysis was employed to discern potential causative molecules. Subsequently, we revisited a previously reported prospective cohort study to reanalyze the relationship among MetS, the identified potential causative molecules, and adverse outcomes in stroke patients [26, 32]. Additionally, potential mediating effects among these factors were explored.

### 6.2 The Study Population and Diagnostic Criteria

The study focused on patients experiencing their first‐ever stroke, with the stroke cohort encompassing cases of spontaneous ICH and IS. ICH was characterized as intracerebral bleeding [35], while IS was defined as localized necrosis or softening of brain tissue resulting from compromised cerebral blood flow, ischemia, and hypoxia [36]. All participants underwent head computed tomography and/or cranial magnetic resonance imaging within 24 h of symptom onset, with diagnoses confirmed by a skilled neuroradiologist [26, 32]. MetS was diagnosed in accordance with the criteria established by the Chinese Diabetes Society in 2004 [30]. The diagnosis of MetS necessitated the presence of three or all of the following four components: (1) central obesity with a BMI ≥ 25 kg/m^2^; (2) elevated blood pressure with readings of ≥140/90 mmHg or confirmed and treated hypertension; (3) elevated Glu levels indicated by fasting plasma Glu ≥ 6.1 mmol/L (110 mg/dL) and/or 2 h plasma Glu ≥ 7.8 mmol/L (140 mg/dL) or a previous diagnosis and treatment for diabetes mellitus; and (4) dyslipidemia denoted by fasting plasma TGs ≥ 1.7 mmol/L (150 mg/dL) and/or fasting HDL‐C < 0.9 mmol/L (35 mg/dL) for men or <1.0 mmol/L (39 mg/dL) for women. Each patient's diagnosis was independently evaluated by two experienced clinicians, with any discrepancies resolved through consensus or consultation with a third reviewer, if necessary.

### 6.3 Establishment of Clinical Outcomes

Primary and secondary clinical endpoints were established to assess outcomes in the study. The primary endpoint was defined as a composite event comprising stroke‐related mortality and stroke recurrence, serving as a measure of adverse outcome poststroke admission. Secondary endpoints included the requirement for surgical intervention, administration of vasoactive medications, and the need for invasive mechanical ventilation, all indicative of adverse events in the clinical course.

### 6.4 Patient Selection and Data Collection Process in the Retrospective Cohort

The retrospective investigation was conducted across three clinical centers within the medical system of Tongji Hospital, Tongji Medical College, Huazhong University of Science and Technology: the Sino‐French New City Branch, Hankou Qiaokou Branch, and Guanggu Branch. This integrated healthcare network maintains an electronic medical record system containing over 60 million discrete clinical records with archival data since 1980. Our analytical dataset comprised patients admitted for stroke‐related conditions between January 2018 and January 2022, systematically extracted from this centralized repository. Exclusion criteria were rigorously applied, eliminating individuals below 18 years of age and pregnant women. The standardized data collection protocol encompassed demographic characteristics (age and gender), behavioral factors (tobacco use and alcohol consumption), physiological parameters (blood pressure measurements and BMI), metabolic profiles (lipid levels and admission Glu concentrations), along with documented comorbidities.

### 6.5 Data Cleaning Process

Data cleaning is an essential aspect of data analysis to ensure data quality and reliability for accurate analyses and valid conclusions [38]. Common issues such as outliers and missing values can significantly impact analysis outcomes. Missing values were observed in the derivation cohort, whereas no missing values were found in the prospective cohort. To address missing values, the “mcartest” command, which conducts Little's test for the Missing Completely at Random (MCAR) assumption and supports diverse missing‐value patterns, is employed (Little's MCAR test *p* value > 0.05). In this study, machine learning algorithms are utilized to interpolate missing data. Specifically, the K Nearest Neighbor (KNN) interpolation method is applied to impute missing values, followed by training a Random Forest regression model to predict the filled data. The Root Mean Square Error metric is calculated to evaluate the model's performance [39]. For outlier treatment, the Winsorizing method is utilized to mitigate the influence of extreme values. Winsorizing involves replacing extreme values in the dataset with values at specified percentiles (1st and 99th percentiles) to minimize their impact on the analysis [40]. Figure S5 depicts the data preprocessing procedure.

### 6.6 Identification and Control of Confounding Factors

In the realm of real‐world clinical data, discrepancies in baseline characteristics are common. To bolster the reliability and consistency of findings, it is vital to adjust for potential confounding variables. This study adopts a multifaceted approach to identify and address confounders. Initially, gender and age were accounted for in correction model 1, aligning with established methodologies in prior literature. Subsequently, in model 2, adjustments were made for gender, age, BMI, smoking, and alcohol consumption based on existing knowledge. Moving forward to corrected model 3, all imbalanced confounders were addressed comprehensively. Furthermore, PSM was utilized to harmonize the two sets of baseline data by screening variables through directed acyclic graphs [41]. Propensity scores for each patient were computed through logistic regression modeling and paired 1:1 with a caliper width set to the standard deviation of 0.1 times the standard deviation of the propensity score. [42]. Standardized mean differences (SMD) were calculated before and after matching to evaluate the balance of variables between the two groups, with an SMD < 0.1 indicating insignificance. Conducting sensitivity analysis using *E*‐value, an essential metric in observational studies for assessing unmeasured confounding [43]. Last, to ensure the robustness of the outcomes, the analysis process was reiterated using the raw data without undergoing data cleaning.

### 6.7 Screening of Disease Molecules by Bioinformatics Analysis

Bioinformatics analyses were utilized to explore shared genes among different components of MetS in conjunction with stroke. Disease‐relevant protein‐coding genes were sourced from the GeneCards database (https://www.Genecards.org) by conducting searches using keywords such as “stroke,” “hypertension,” “diabetes,” and “obesity” [44, 45]. Subsequently, the pertinent protein‐coding targets associated with these diseases were extracted and compiled into a excel table (Tables S8–S11). The relevance of the gene targets to cerebral ischemia–reperfusion was assessed based on the relevance scores assigned in the GeneCards database, with a higher score indicating closer relevance. Genes with GeneCards Inferred Functionality Scores (GIFtS) equal to or greater than 50 were identified, extracted, and tabulated in an Excel sheet (stroke genes: *n* = 3206; hypertension genes: *n* = 3938; obesity genes: *n* = 3546; diabetes genes: *n* = 4349) [46]. Venn diagrams were employed to display the intersections between the four diseases under investigation (Table S12). The identified genes underwent further analysis to assess their molecular functions and pathway associations using Gene Ontology (GO) and Kyoto Encyclopedia of Genes and Genomes (KEGG) enrichment analyses, with statistical significance set at a *p* value < 0.05 [47].

The KKS comprises an array of potassium‐lowering kinases (kallikreins) and peptides (kinins) primarily associated with vasodilation, anticoagulation, and anti‐inflammatory activities [48]. KKS‐associated gene sets were compiled from pertinent literature sources (Table S13). The essential genes within the KKS were scrutinized through the examination of the PPI network [49]. To pinpoint specific target genes relevant to the KKS pathway in relation to stroke and MetS, the gene set intersection identified via GeneCards and KKS‐associated genes was employed as the final target genes. Through the application of Friends analysis, gene interaction networks were constructed, and the significance of each gene was evaluated using network topology parameters (Table S14).

Retrieve mRNA expression data for key components of the MetS including hypertension, diabetes, and obesity, along with relevant clinical information from the GEO database using the datasets GSE18897, GSE30122, and GSE74144 [50, 51, 52]. The annotation and preprocessing of the microarray data were conducted using the “GEOquery” and “BiocManager” packages [33]. The verification of gene expression levels within each of the gene sets was performed.

### 6.8 Data Sources and Study Design for Validation Cohort

A national multicenter prospective cohort was employed as the validation cohort for the prior prospective study, for which comprehensive details regarding inclusion and exclusion criteria, as well as study implementation, have been previously documented [26, 32]. The study cohort was derived from a collaborative initiative involving five tertiary cardiovascular institutions in China: Fuwai Hospital, Chinese Academy of Medical Sciences and Peking Union Medical College (Beijing); The First Affiliated Hospital of Xi'an Jiaotong University (Xi'an); Tongji Hospital, Huazhong University of Science and Technology (Wuhan); Tianjin Cardiovascular Institute (Tianjin); and Daping Hospital, Army Medical University (Chongqing). Data collection was conducted through a standardized prospective observational protocol implemented from November 2000 to November 2001 across all participating centers. This coordinated research protocol ensured uniformity in data acquisition procedures across the municipalities of Beijing, Tianjin, Wuhan, Xi'an, and Chongqing.

This prospective cohort comprised 1268 patients experiencing their first‐ever stroke, with a median follow‐up duration of 5 years (range, 4–5.5 years). Individuals with concurrent chronic kidney disease or chronic respiratory ailments were excluded from the study. Blood samples were collected from these patient's postacute stroke phase (within 1 month to 1 year after onset). Upon collection, the samples were promptly placed in precooled centrifuge tubes and centrifuged under controlled temperatures to separate clear plasma from cellular precipitates. Subsequently, the samples were stored in an ultra‐low temperature freezer at −80°C. The plasma KLK1 levels were quantified using a double antibody sandwich biotin affinity enzyme‐linked immunosorbent assay kit. Secondary analyses were conducted using the clinical data, with detailed descriptions of the sample collection and assay protocols outlined in previous publications [26].

### 6.9 Statistical Analysis

Statistical analyses and graphical visualization in this study were conducted using R software (version 4.3.0), anaconda3 (Anaconda, Inc), and Stata 17.0 software (Stata Corp). Descriptive statistics were determined based on data normality, presented as mean ± standard deviation or median (upper and lower quartiles) for continuous variables, and as frequencies and percentages for categorical variables. Figure S6 displays the distribution and correlation of continuous variables in the dataset. Two‐group comparisons of continuous variables were assessed using the *t*‐test for normally distributed data and Welch's *t*‐test for data conforming to normal distribution but not to chi‐square test. In cases of non‐normally distributed data, the Wilcox test was employed. Categorical variable comparisons between groups were analyzed using the chi‐square test with a significance level of *p *< 0.05.

Proportional hazards tests were carried out using the “estat phtest” command in Stata. Nonlinear correlations between various variables and the outcome event were assessed through a Cox proportional hazards regression model utilizing restricted cubic spline with four knots at the 5th, 35th, 65th, and 95th percentiles. The “utest” command is utilized to assess whether there exists a U‐shaped or inverted U‐shaped relationship between the explanatory and outcome variables [53]. The Boruta algorithm was applied to ascertain the significance of different features in the Cox proportional hazards regression model regression model incorporating covariates (doTrace = 2, ntree = 500, *p* value = 0.01) [54]. Subsequently, the SHAP value theory was employed to elucidate the impact of KLK1 levels on the poor prognosis of stroke patients within the Cox proportional hazards regression model regression models incorporating covariates [55]. The calculation of SHAP values and the implementation of The Boruta algorithm are achieved through the R software. The “medeff” method was utilized for analyzing mediating effects, providing estimates for various data types and accounting for binary outcomes and mediators.

## Author Contributions

H.R. and X.R. was responsible for statistical analyses and the initial draft of the manuscript. Q.Z., T‐T.X., D‐Y.D., and F.L. conducted data cleaning and contributed to the study design, data analysis, and interpretation. Q.Z., D‐W.W., and S‐S.L. contributed to revising the manuscript critically for intellectual content and approved the final version for publication. All authors reviewed the manuscript critically for intellectual content and have read and approved the final manuscript.

## Ethics Statement

The study received approval from the Institutional Review Board of Tongji Hospital, Tongji Medical College, Huazhong University of Science and Technology (Wuhan, China; Approval No. TJ‐IRB20230830), and followed the guiding principles outlined in the Helsinki Declaration. The clinical trial is registered with ClinicalTrials.gov (NCT06165107). Data for this study comprise two cohorts. Written informed consent was obtained from all participants in the prospective cohort. For the retrospective analysis, informed consent was waived following ethics committee approval due to the observational nature of the study, as only deidentified data were accessed via secure servers. All participant information has been anonymized.

## Conflicts of Interest

The authors declare no conflicts of interest.

## Funding

This work was supported by grants from Health Ministry of China, National 863 project (No. 2006AA02A406), and 973 project (No. 2007CB512004). This study was supported by funding from the National Natural Science Foundation of China (Grant No. 82271358, 81100864), Natural Science Foundation of Hubei Province (2024AFB642, 2025AFB609) and Technology, and the Talent Project of Public Health in Hubei Province (Grant No. 2022SCZ048) and the National Basic Research Program of China (Grant No. 2007CB512004).

## Supporting information




**Supporting File 1**: mco270506‐sup‐0001‐SuppMat.docx

**Supporting File 2**: mco270506‐sup‐0001‐tablesS1‐S14.xlsx

## Data Availability

The data and code used in this study could be obtained from the corresponding author upon reasonable request.
