# Supplementary material for: Tissue Kallikrein as a Mediator in Stroke Outcomes among Patients With Metabolic Syndrome: A Multicenter Study Integrating Big Data and Biomarkers
Source: MedComm (2020). 2025 Nov 26;6(12):e70506. doi: 10.1002/mco2.70506 (PMC12657629; doi:10.1002/mco2.70506)
Supplement: Supplementary file 1 — Supporting File 1: mco270506‐sup‐0001‐SuppMat.docx [file MCO2-6-e70506-s002.docx]

**Supplementary Material**

**KLK1-Mediated Impacts of Metabolic syndrome on Stroke Prognosis: A Multicenter Observational Study**

Hang Ruan^1,2^ ^*^, Xiao Ran ^1,2*^, Ting-ting Xu^1,2^, Da-yong Li^1,2^, Shu-sheng Li ^1,2#^, Dao-Wen Wang^3#^, Qin Zhang^4#^

^1^Department of Critical-care Medicine, Tongji Hospital, Tongji Medical College, Huazhong University of Science and Technology, Wuhan, 430030, China.

^2^Department of Emergency Medicine, Tongji Hospital, Tongji Medical College, Huazhong University of Science and Technology, Wuhan, 430030, China.

^3^The Institute of Hypertension and Department of Internal Medicine, Tongji Hospital, Tongji Medical College, Huazhong University of Science and Technology, Wuhan 430030, China

^4^Department of Anesthesiology, Hubei Key Laboratory of Geriatric Anesthesia and Perioperative Brain Health, and Wuhan Clinical Research Center for Geriatric Anesthesia, Tongji Hospital, Tongji Medical College, Huazhong University of Science and Technology, Wuhan, 430030, China.

^*^ These authors contributed equally to this work.

**#Corresponding authors:**

Qin Zhang: 1095# Jiefang Ave., Wuhan 430030 People’s Rep. of China; Tel. 86-15717154768; Email: qzhang8@tjh.tjmu.edu.cn

Dao Wen Wang: 1095# Jiefang Ave., Wuhan 430030 People’s Rep. of China; Email: dwwang@tjh.tjmu.edu.cn

Shu-sheng Li: 1095# Jiefang Ave., Wuhan 430030 People’s Rep. of China; Tel. +86-13971086498; Email: Shushengli16@sina.com

Contents

[Figure S1 3](#_Toc199794323)

[Figure S2 4](#_Toc199794324)

[Figure S3 5](#_Toc199794325)

[Figure S4 6](#_Toc199794326)

[Figure S5 7](#_Toc199794327)

[Figure S6 8](#_Toc199794328)

[STROBE Statement 9](#_Toc199794336)

# Figure S1


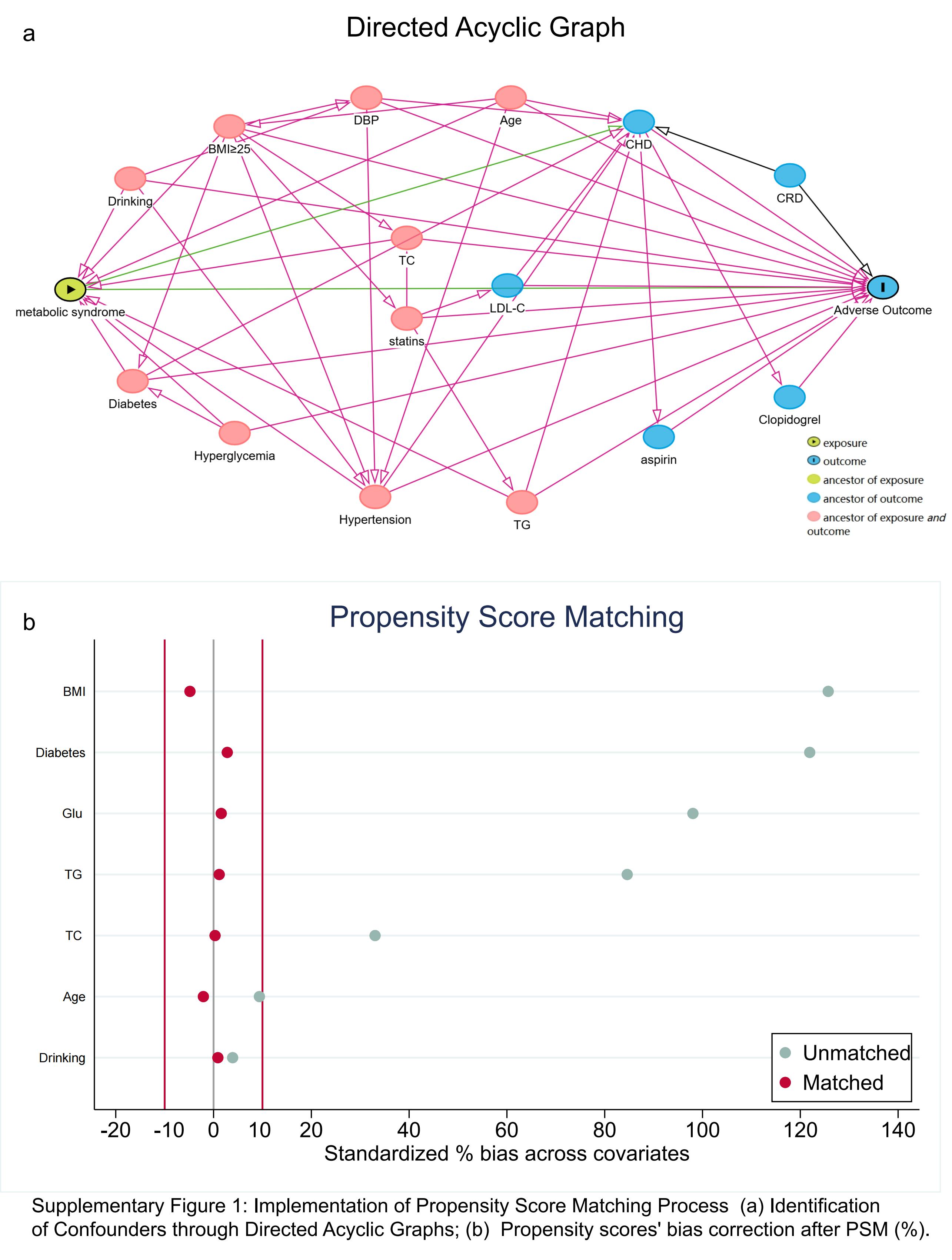


# Figure S2


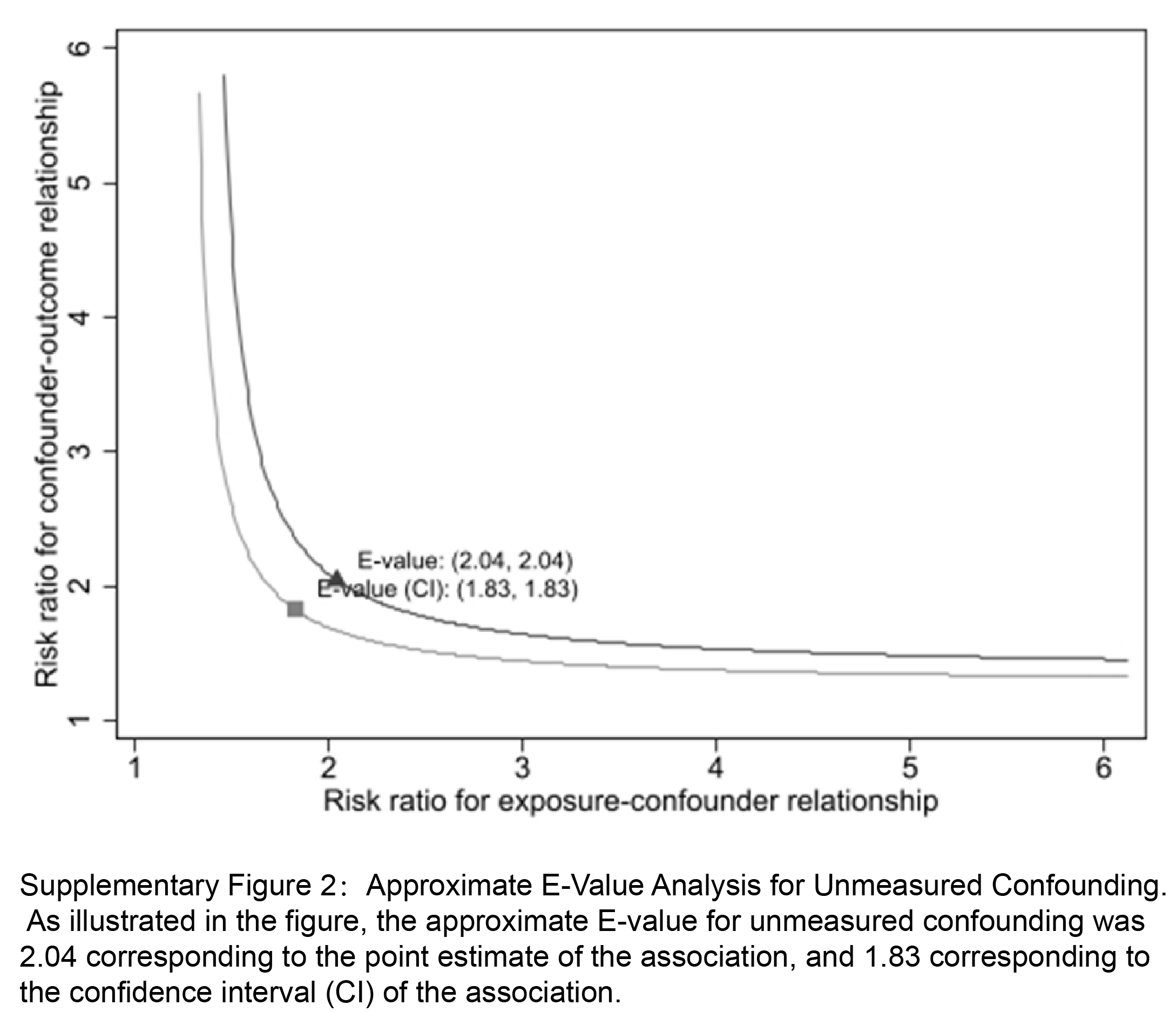


# Figure S3


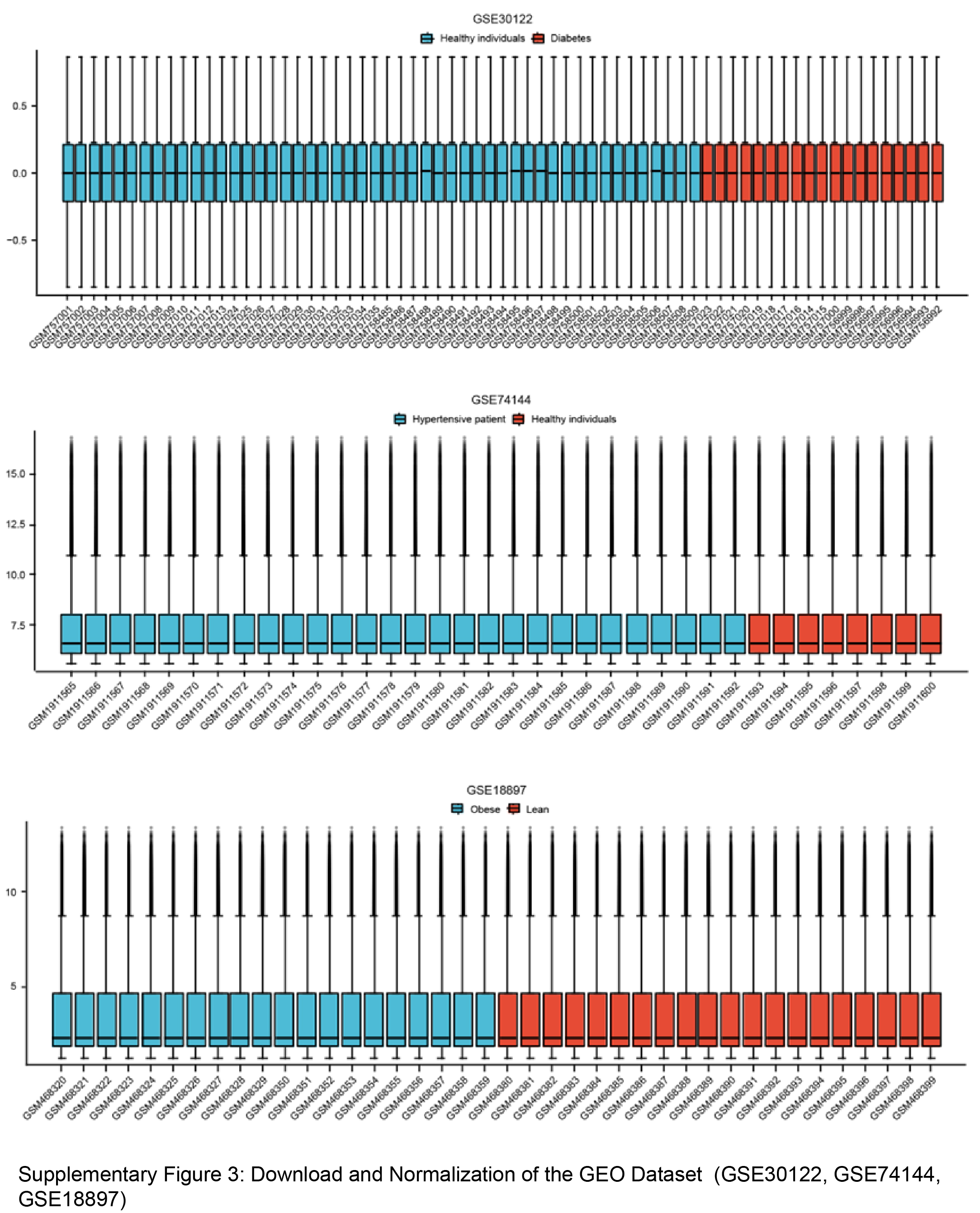


# Figure S4


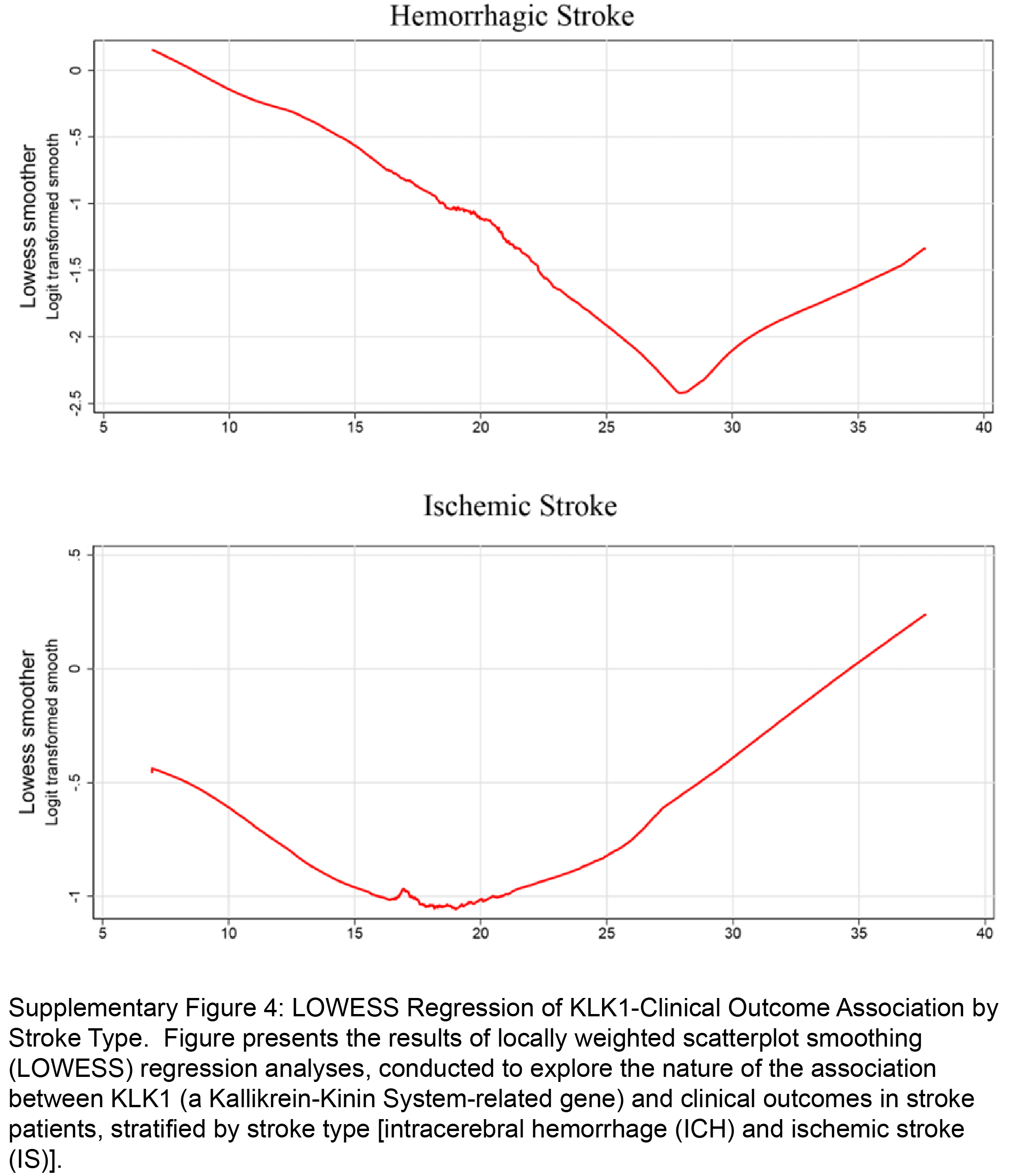


# Figure S5


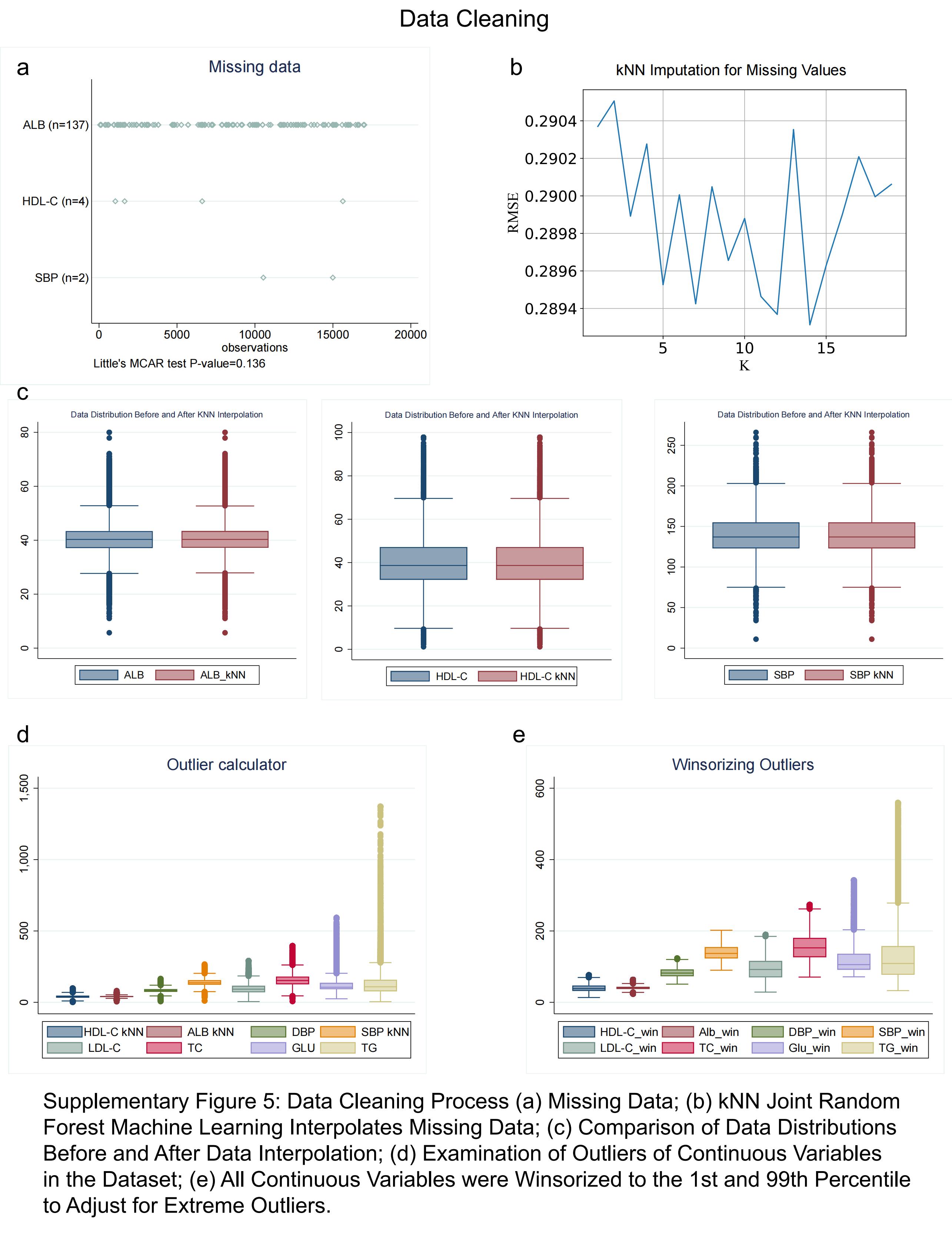


# Figure S6


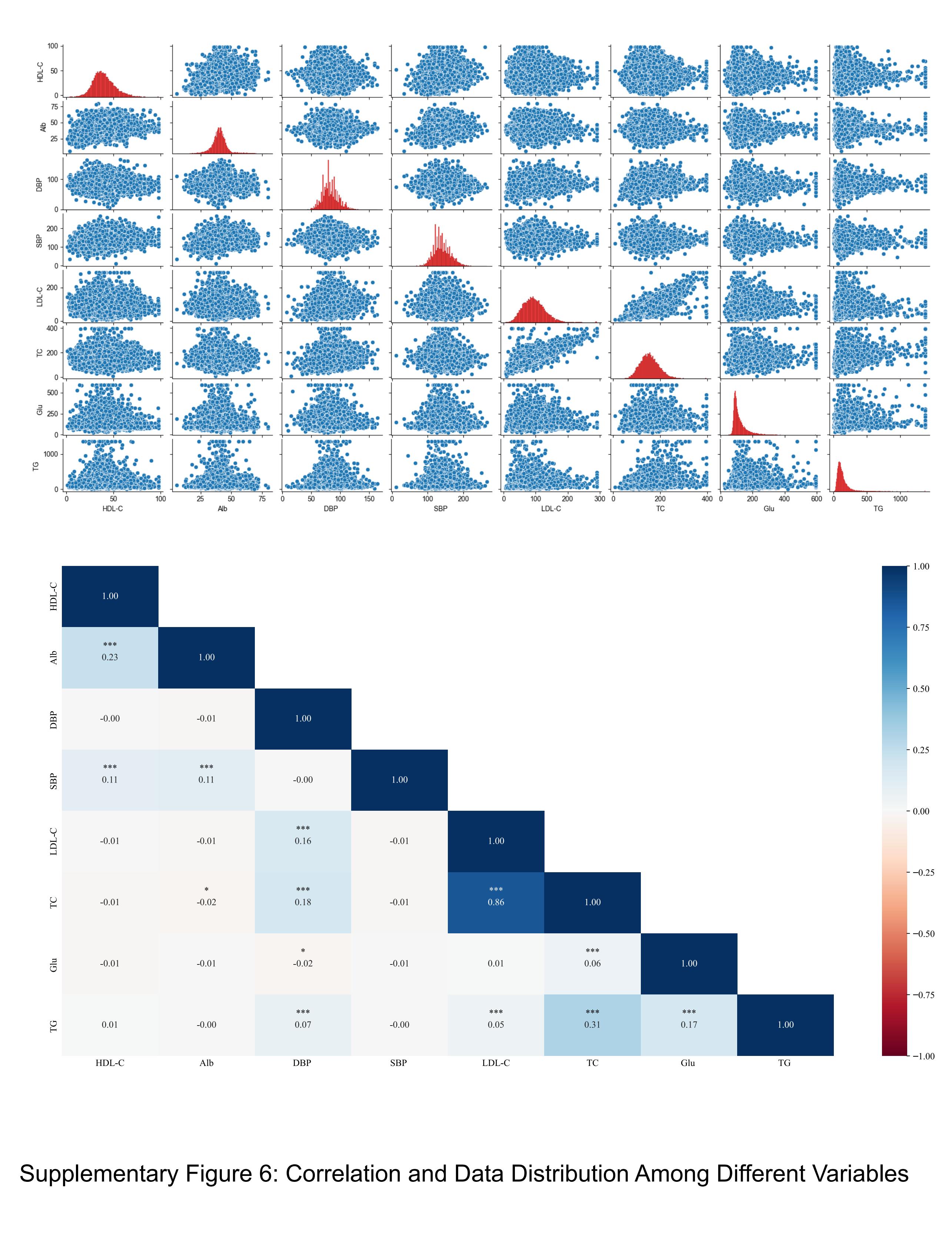


# STROBE Statement—checklist of items that should be included in reports of observational studies

|  | **Item No.** | **Recommendation** | **Page  No.** | **Relevant text from manuscript** |
| --- | --- | --- | --- | --- |
| **Title and abstract** | 1 | (*a*) Indicate the study’s design with a commonly used term in the title or the abstract | 1 | a multicentre observational study |
|  |  | (*b*) Provide in the abstract an informative and balanced summary of what was done and what was found | 2 | See manuscript for more details |
| **Introduction** | | | |  |
| Background/rationale | 2 | Explain the scientific background and rationale for the investigation being reported | 3 | examine the effect of MetS on the prognosis of stroke patients |
| Objectives | 3 | State specific objectives, including any prespecified hypotheses | 4 | investigate tissue kallikrein (KLK1) as a potential mediator of this impact. |
| **Methods** | | | |  |
| Study design | 4 | Present key elements of study design early in the paper | 4 | MetS, KLK1, stroke-related mortality and recurrence |
| Setting | 5 | Describe the setting, locations, and relevant dates, including periods of recruitment, exposure, follow-up, and data collection | 5-6 | See manuscript for more details |
| Participants | 6 | (*a*) *Cohort study*—Give the eligibility criteria, and the sources and methods of selection of participants. Describe methods of follow-up  *Case-control study*—Give the eligibility criteria, and the sources and methods of case ascertainment and control selection. Give the rationale for the choice of cases and controls  *Cross-sectional study*—Give the eligibility criteria, and the sources and methods of selection of participants | 5-6 | stroke Patients |
|  |  | (*b*) *Cohort study*—For matched studies, give matching criteria and number of exposed and unexposed  *Case-control study*—For matched studies, give matching criteria and the number of controls per case | 5-6 | Cohort study |
| Variables | 7 | Clearly define all outcomes, exposures, predictors, potential confounders, and effect modifiers. Give diagnostic criteria, if applicable | 5-6 | See manuscript for more details |
| Data sources/ measurement | 8* | For each variable of interest, give sources of data and details of methods of assessment (measurement). Describe comparability of assessment methods if there is more than one group | 5-6 | Tongji hospital and GEO database |
| Bias | 9 | Describe any efforts to address potential sources of bias | 8 | PSM, sensitivity analyses |
| Study size | 10 | Explain how the study size was arrived at |  | not applicable |
| Quantitative variables | 11 | Explain how quantitative variables were handled in the analyses. If applicable, describe which groupings were chosen and why | 7 | age and gender, lifestyle habits like smoking and drinking, blood pressure readings, lipid profiles, admission glucose levels, BMI, and comorbidities. |
| Statistical methods | 12 | (*a*) Describe all statistical methods, including those used to control for confounding | 7 | PSM and Multivariate analysis |
|  |  | (*b*) Describe any methods used to examine subgroups and interactions | 8 | Subgroup analysis |
|  |  | (*c*) Explain how missing data were addressed | 7 | KNN and winsorizing |
|  |  | (*d*) *Cohort study*—If applicable, explain how loss to follow-up was addressed  *Case-control study*—If applicable, explain how matching of cases and controls was addressed  *Cross-sectional study*—If applicable, describe analytical methods taking account of sampling strategy | 5-6 | Cohort study |
|  |  | (*e*) Describe any sensitivity analyses | 7-8 | the analysis process was reiterated using the raw data without undergoing data cleaning. |
| **Results** | | | | |
| Participants | 13* | (a) Report numbers of individuals at each stage of study—eg numbers potentially eligible, examined for eligibility, confirmed eligible, included in the study, completing follow-up, and analysed | 12 | Consider use of a flow diagram |
|  |  | (b) Give reasons for non-participation at each stage | 12 | See manuscript for more details |
|  |  | (c) Consider use of a flow diagram | 12 | Figure1 |
| Descriptive data | 14* | (a) Give characteristics of study participants (eg demographic, clinical, social) and information on exposures and potential confounders | 12 | Table1 |
|  |  | (b) Indicate number of participants with missing data for each variable of interest | 12 | Table1 |
|  |  | (c) *Cohort study*—Summarise follow-up time (eg, average and total amount) | 12 | median follow-up duration of 5 years (range, 4-5.5 years) |
| Outcome data | 15* | *Cohort study*—Report numbers of outcome events or summary measures over time | 12 | Table1 |
|  |  | *Case-control study—*Report numbers in each exposure category, or summary measures of exposure |  |  |
|  |  | *Cross-sectional study—*Report numbers of outcome events or summary measures |  |  |
| Main results | 16 | (*a*) Give unadjusted estimates and, if applicable, confounder-adjusted estimates and their precision (eg, 95% confidence interval). Make clear which confounders were adjusted for and why they were included | 13 | Figure2 |
|  |  | (*b*) Report category boundaries when continuous variables were categorized | 13 | Figure2 |
|  |  | (*c*) If relevant, consider translating estimates of relative risk into absolute risk for a meaningful time period |  | not applicable |
| Other analyses | 17 | Report other analyses done—eg analyses of subgroups and interactions, and sensitivity analyses | 14 | Figure2, E-VALUE |
| **Discussion** | | | | |
| Key results | 18 | Summarise key results with reference to study objectives | 16-17 | See manuscript for more details |
| Limitations | 19 | Discuss limitations of the study, taking into account sources of potential bias or imprecision. Discuss both direction and magnitude of any potential bias | 21 | See manuscript for more details |
| Interpretation | 20 | Give a cautious overall interpretation of results considering objectives, limitations, multiplicity of analyses, results from similar studies, and other relevant evidence | 16-19 | See manuscript for more details |
| Generalisability | 21 | Discuss the generalisability (external validity) of the study results | 20 | See manuscript for more details |
| **Other information** | |  | | |
| Funding | 22 | Give the source of funding and the role of the funders for the present study and, if applicable, for the original study on which the present article is based | 22 | Tongji hospital, 863 program |

*Give information separately for cases and controls in case-control studies and, if applicable, for exposed and unexposed groups in cohort and cross-sectional studies.

**Note:** An Explanation and Elaboration article discusses each checklist item and gives methodological background and published examples of transparent reporting. The STROBE checklist is best used in conjunction with this article (freely available on the Web sites of PLoS Medicine at http://www.plosmedicine.org/, Annals of Internal Medicine at http://www.annals.org/, and Epidemiology at http://www.epidem.com/). Information on the STROBE Initiative is available at www.strobe-stateme.
